# Advanced Age May Not Be an Absolute Contraindication for Radical Nephroureterectomy in Patients with Upper Tract Urothelial Carcinoma: A Single-Center Case Series and a Systematic Review with Meta-Analyses

**DOI:** 10.3390/jcm12237273

**Published:** 2023-11-24

**Authors:** Jianjun Ye, Qiyou Wu, Xinyang Liao, Lei Zheng, Qiang Wei, Yige Bao

**Affiliations:** 1 Department of Urology and Institute of Urology, West China Hospital, Sichuan University, Chengdu 610041, China; yejianjun2022@stu.scu.edu.cn (J.Y.); wuqiyou@stu.scu.edu.cn (Q.W.); liaoxinyang@wchscu.edu.cn (X.L.); urozhenglei@163.com (L.Z.); 2 West China School of Medicine, Sichuan University, Chengdu 610041, China

**Keywords:** advanced age, octogenarians, upper urinary tract urothelial carcinoma, radical nephroureterectomy

## Abstract

Objective: This study aims to investigate whether advanced age is an absolute contraindication for radical nephroureterectomy (RNU) in patients with upper tract urothelial carcinoma (UTUC) through a single-center case series and a systematic review with meta-analysis. Materials and methods: In the single-center case series, 588 UTUC patients who underwent RNU between May 2003 and June 2019 in West China Hospital were enrolled, and cancer-specific survival (CSS) was the primary outcome of interest. In the systematic review with meta-analysis, PubMed, Scopus, Embase, and Cochrane databases were systematically searched for related articles for further analysis. The endpoints for meta-analyses were overall survival (OS) and CSS. Results: The single-center case series included 57 (9.7%) octogenarians. The CSS of octogenarians after RNU was comparable to that of younger people. Advanced age (≥80) was not an independent risk factor for poor CSS (HR, 1.08; 95% CI, 0.48, 2.40). In a systematic review with meta-analysis, the cut-off value of advanced age is 70, and the results showed that advanced age was associated with inferior OS (pooled HR, 1.55; 95% CI, 1.29, 2.01) and CSS (pooled HR, 1.37; 95% CI, 1.08, 1.65). However, the subgroup analysis of countries found no positive correlation between advanced age and CSS (pooled HR, 1.33; 95% CI 0.92, 1.74) in Chinese. Conclusions: Advanced age may no longer be an absolute contraindication for RNU. RNU can be safely and effectively performed on UTUC patients of advanced age after a comprehensive presurgical evaluation.

## 1. Introduction

Urothelial carcinoma (UC) is the fourth most common malignancy in developed countries, and upper tract urothelial carcinoma (UTUC) only accounts for 5–10% of UC, with an estimated annual incidence of approximately 2 people per 100,000 [1,2]. Radical nephroureterectomy (RNU), with or without adjuvant therapy, serves as the gold standard treatment for high-risk organ-confined UTUC, providing durable local control and maximizing survival expectations [2]. Moreover, as upper tract urothelial carcinoma (UTUC) is most common in patients aged 70–90 years, the number of UTUC patients of advanced age worldwide is gradually increasing in the context of global aging.

Great advancements in anesthesia, surgical techniques (especially laparoscopy and robotics), and nursing during hospitalization have contributed to the theoretical and practical foundation of operations on elderly patients [3,4,5]. Recently, a growing number of studies have reported similar survival outcomes between younger and older patients after surgery [6,7]. However, whether RNU is a suitable and promising choice for patients of advanced age is heavily debated and remains unclear.

Therefore, the present study aims to investigate whether advanced age is an absolute contraindication for RNU by reporting the survival outcomes of UTUC patients with advanced age after RNU in Southwest China and placing our findings within the context of the literature by performing a systematic review and meta-analysis of studies on the topic of age and UTUC prognosis after RNU.

## 2. Methods and Materials

### 2.1. Single-Center Case Series

#### 2.1.1. Patients and Data

**Figure 1:**
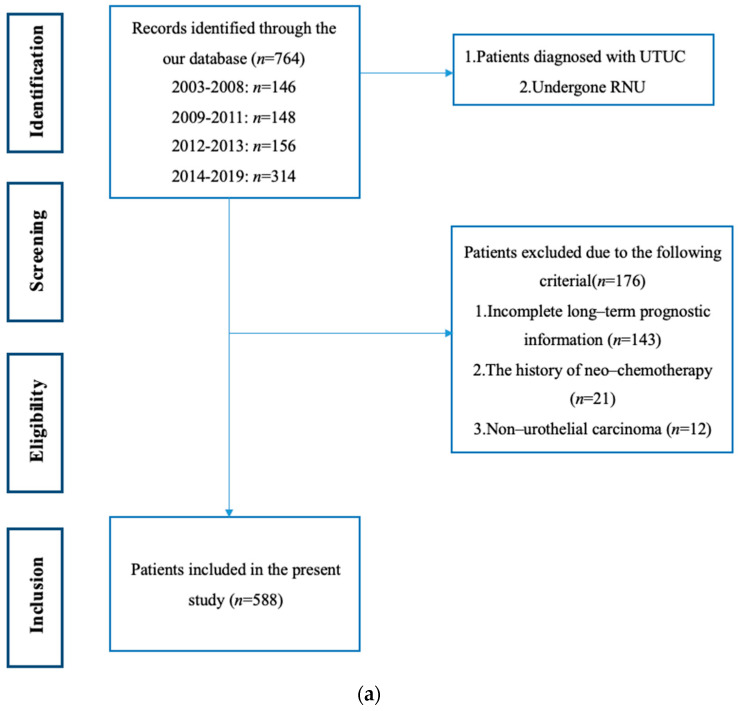

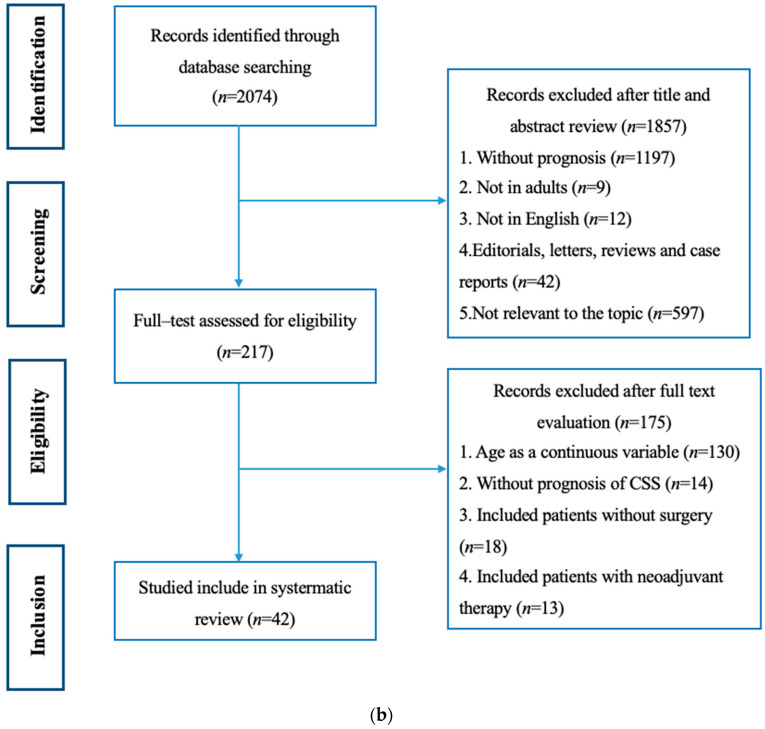
(**a**) The specific screening flow chart of included patients. (**b**) The specific screening flow chart of included studies.

Our study was approved by the Ethics Committee on Biomedical Research, West China Hospital of Sichuan University (2021–1209), and the informed consent was allowed to be waived because of the retrospective nature, anonymous patients, and data. We retrospectively collected UTUC patients who had undergone RNU between May 2003 and June 2019 in the West China Hospital of Sichuan University. We excluded those with incomplete long-term prognostic information, those with non-urothelial carcinoma confirmed by RNU pathology, and we also excluded those who had a previous neoadjuvant therapy history. Ultimately, 588 patients were eligible for inclusion in further analysis (Figure 1a).

We collected clinical and pathological data from our hospital database. Clinical data included chronological age in the year of surgery, gender, body mass index (BMI), hemoglobin and albumin within 1 week before RNU, the American Society of Anesthesiologists (ASA) score, the history of adjuvant systemic chemotherapy, and surgical techniques. Pathological data included tumor location, tumor size, pathological tumor stage and grade, lymph node status, lymphovascular invasion (LVI), tumor architecture and multifocality, and surgical margin. The pathological tumor stage and grade were both derived by an independent review of RNU specimens by our professional urogenital pathologists, according to the American Joint Committee on Cancer (AJCC) tumor-node-metastasis (TNM) staging system and the WHO/ISUP recommendation grading system, respectively.

#### 2.1.2. Follow-Up and Endpoints

Approximately 2 to 3 weeks after RNU is the rational time for first follow-up, at which time the pathological reports of the RNU specimen, including the general report and immunohistochemical report, have been completed. The main project was to analyze the nature of the primary tumor and make individual therapy plans. Generally, the protocol of subsequent follow-up was in accordance with the European Association of Urology guidelines. The patients were followed up every 3 months in the first year, every 6 months in the second year, and then once every 2 years if there were no recurrence or uncommon symptoms. The routine contents of follow-up included a physical examination, routine blood and urine tests, a contrast-enhanced computed tomography scan of the chest and abdomen, and cystoscopy.

Cancer-specific survival (CSS), defined as the time from RNU to death related to UTUC, and cancer-specific mortality were included in our study as the endpoints.

#### 2.1.3. Statistical Analysis

Analysis was performed using the software packages R 4.3.2 (http://www.R-project.org; accessed on 21 September 2023, The R Foundation, Boston, MA, USA) and EmpowerStats 4.0 (http://www.empowerstats.com; accessed on 21 September 2023 X and Y Solutions, Inc., Boston, MA, USA). Continuous variables were expressed as medians with interquartile ranges (IQRs), and categorical variables were expressed as numbers with percentages. Significant differences between groups were determined by the χ^2^ test and Mann–Whitney U test. CSS was calculated using Kaplan–Meier analysis, and the log-rank test was used for comparison. Cox proportional hazard models were used to investigate the associations of clinicopathological information with CSS by calculating HRs (95% CI). All statistical assessments were two-tailed and considered statistically significant at *p* < 0.05.

### 2.2. Systematic Review and Meta-Analysis

The conduct and reporting form of this meta-analysis were in accordance with the Meta-Analysis extension of the Preferred Reporting Items for Systematic Reviews and Meta-Analyses (PRISMA-NMA) reporting guidelines [8]. The PRISMA checklist was provided in Supplementary Table S1.

#### 2.2.1. Search Strategy and Selection Criteria

The PubMed, Scopus, Embase, and Cochrane databases were systematically searched for related articles. The predefined search terms are “UTUC,” “upper tract urothelial carcinoma,” “upper tract urinary carcinoma,” “urothelial carcinoma,” “renal pelvis and ureter tumor,” “radical nephroureterectomy” and “RNU”. Synonyms were combined by the Boolean operator (OR), and paratactic terms were combined by the Boolean operator (AND).

Population, intervention, comparison, outcomes, and study (PICOS) principles were used during the screening process [9], where PICOS stands for: population (P)—patients diagnosed with UTUC; intervention (I)—RNU surgery; comparison (C)—octogenarians and younger patients; outcomes (O)—cancer-specific mortality and CSS; and study designs (S)—retrospective comparative studies. The exclusion criteria were as follows: (a) age was regarded as a continuous variable; (b) not all patients had received RNU; (c) included patients with neoadjuvant therapy; and (d) there was no specific information on CSS. The specific screening flow chart is shown in Figure 1b.

#### 2.2.2. Data Extraction and Quality Assessment

Two reviewers (JJY and QYW) independently extracted the following data, and disagreements were resolved through consensus: (1) first author’s name, (2) publication year, (3) country, (4) retrospective period, (5) number of patients, (6) median age, (7) cut-off value of age, (8) median follow-up time, (9) RNU surgical method, (10) use of neo-/adjuvant therapy, and (11) survival outcomes of overall survival (OS) and CSS.

The risk of bias was assessed independently by two investigators (JJY and XYL) using the Newcastle Ottawa Scale (NOS). The risk of bias was grouped into three categories, including “high quality” (points range: 7–9), “moderate quality” (points range: 4–6), and “low quality” (points range: 0–3). The disagreement was addressed by consulting with the co-authors.

#### 2.2.3. Data Consolidation

We completed the complicated data synthesis process using StataMP 17 software. The HRs and 95% CIs of OS and CSS were obtained and pooled for analysis. The heterogeneity among studies was evaluated by the I^2^ value. If the I^2^ value was greater than 50%, the heterogeneity was considered to be significant, and a random effects model was used. Egger’s and Begg’s tests were utilized to evaluate the publication bias of our study. Subgroup analyses were performed on regions as well. The significant cut-off values of the *p* value were 0.05 for pooled results and 0.10 for publication bias.

## 3. Results

### 3.1. Single-Center Case Series

#### 3.1.1. Baseline Characteristics of Total and Grouped Patients

A total of 588 patients were included in a single-center case series, with a male-to-female ratio of 322:266 and a median age of 68 (IQR: 60–75) years. A total of 292 (49.9%) patients were in high stage (T3/T4), and 435 (74.6%) patients were in high grade. The final pathological reports showed that lymph node metastasis (LNM), lymphovascular invasion, tumor necrosis, papillary architecture, and positive surgical margins occurred in 59 (9.6%), 92 (15.7%), 45 (7.7%), 319 (54.9%), and 49 (8.4%) cases, respectively.

There were 57 patients in the octogenarian group, with a median age of 83 (IQR: 81–85). There were 531 patients in the younger group, with a median age of 67 (IQR: 59–73). The octogenarian group had a significant association with lower BMI (*p* = 0.042), lower albumin (*p* = 0.019), a higher ASA score (*p* < 0.001), and an earlier tumor stage (*p* = 0.023). No significant differences were found for other characteristics (all *p* > 0.05).

Detailed clinicopathologic and laboratory characteristics are given in Table 1.

#### 3.1.2. Survival Outcomes and Cox Regression

**Figure 2:**
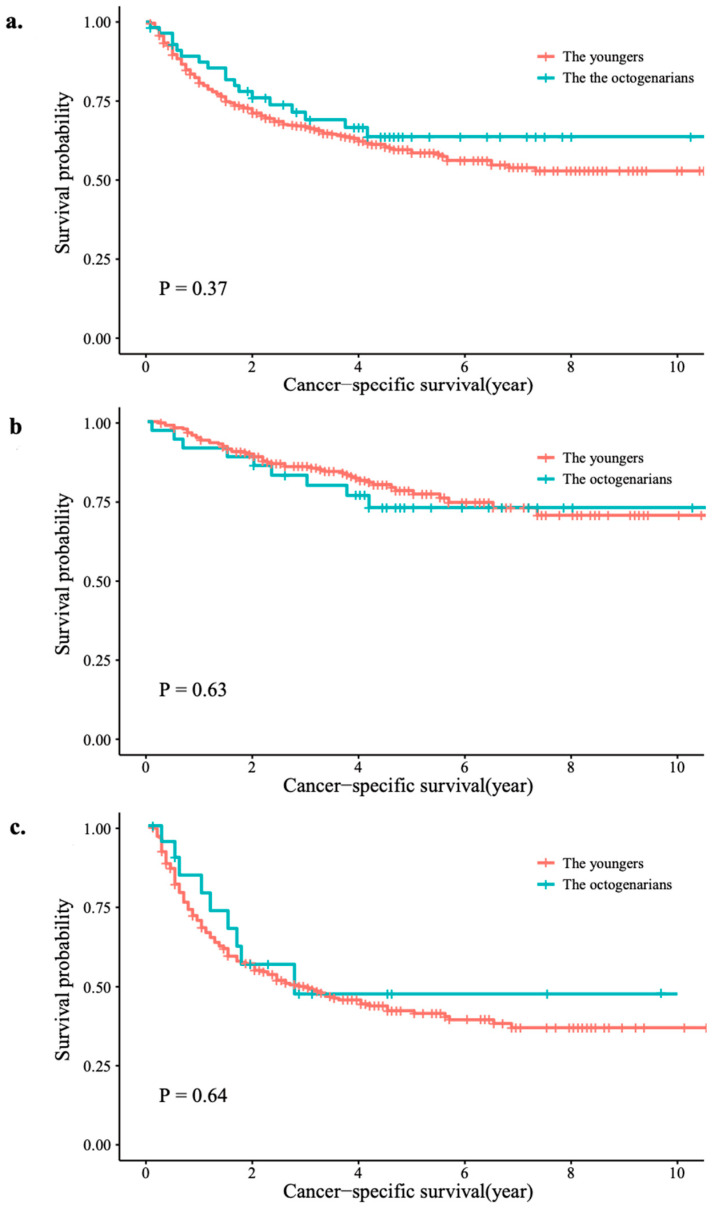
Survival rates for the octogenarians and younger patients: (**a**) in the whole group; (**b**) tumor stage < T3; (**c**) tumor stage ≥ T3.

The median follow-up time was 59 months (IQR: 32–83). During this period, 220 (37.4%) patients died from UTUC. The Kaplan–Meier plots showed that, compared with the younger group, the patients in the octogenarian group showed no significant difference in CSS (*p* = 0.37) (Figure 2a). Similar results were also found in the subgroup analysis of tumor stage (*p* = 0.63 for pT < 3; *p* = 0.64 for pT ≥ 3) (Figure 2b,c).

As shown in Table 2, tumors with advanced stage and grade, tumor size greater than 3, papillary architecture, LNM, positive surgical margin, ureter location, as well as patients with anemia, lower albumin, and without adjuvant systemic chemotherapy history were associated with poor CSS in the univariate Cox analysis (all *p* < 0.05). Then, to include as many clinical indicators as possible, we included all variables whose *p* values were less than 0.10 in the multivariate Cox regression, and the results indicated that tumor stage, size, site, grade, and lymph node status were independently associated with CSS (all *p* < 0.05). However, age was not a significant risk factor in univariate or multivariate analysis (HR, 0.80, 95% CI, 0.50, 1.30, *p* = 0.376; and HR, 1.08, 95% CI, 0.48, 2.40, *p* = 0.853, respectively).

### 3.2. Systematic Review and Meta-Analysis

#### 3.2.1. Systematic Review

The systematic search yielded a total of 2074 studies, of which 42 were found to be eligible after full-text evaluation (Figure 1b). The main characteristics of the included studies are summarized in Table 3. Among the 42 studies, the sample sizes ranged from 24 to 3544 patients, and a total of 22,490 UTUC patients after RNU were included. These studies were published between 2006 and 2023, and the patient recruitment periods ranged from 1987 to 2021. The median age ranged from 61.0 to 74.5 years, and the median follow-up time was between 18.0 and 82.8 months.

Interestingly, nearly all of the included studies (35/42, 83.3%) were Asian studies; three studies were global. It is noteworthy that only five studies (5/42, 11.9%) focused on the influence of the specific cut-off value of age on survival outcomes, while in others, age was just a baseline characteristic and happened to be a categorical variable. Although the age variable was noncontinuous in all the included studies, there was still a large difference among studies that emphasized dichotomous or multicategorical variables and their cut-off values.

#### 3.2.2. Quality Assessment

NOS was used to evaluate the quality of the included studies, and the results are shown in Supplementary Table S2. High quality was determined to be high in 39 studies, moderate in 3 studies, and no study was marked as low quality.

#### 3.2.3. Meta-Analysis

Considering the aim of the present study and the number limitation of the included studies with the same age cut-off value, we finally chose 70 as a relatively appropriate value, and the corresponding studies were subjected to meta-analysis.

**Figure 3:**
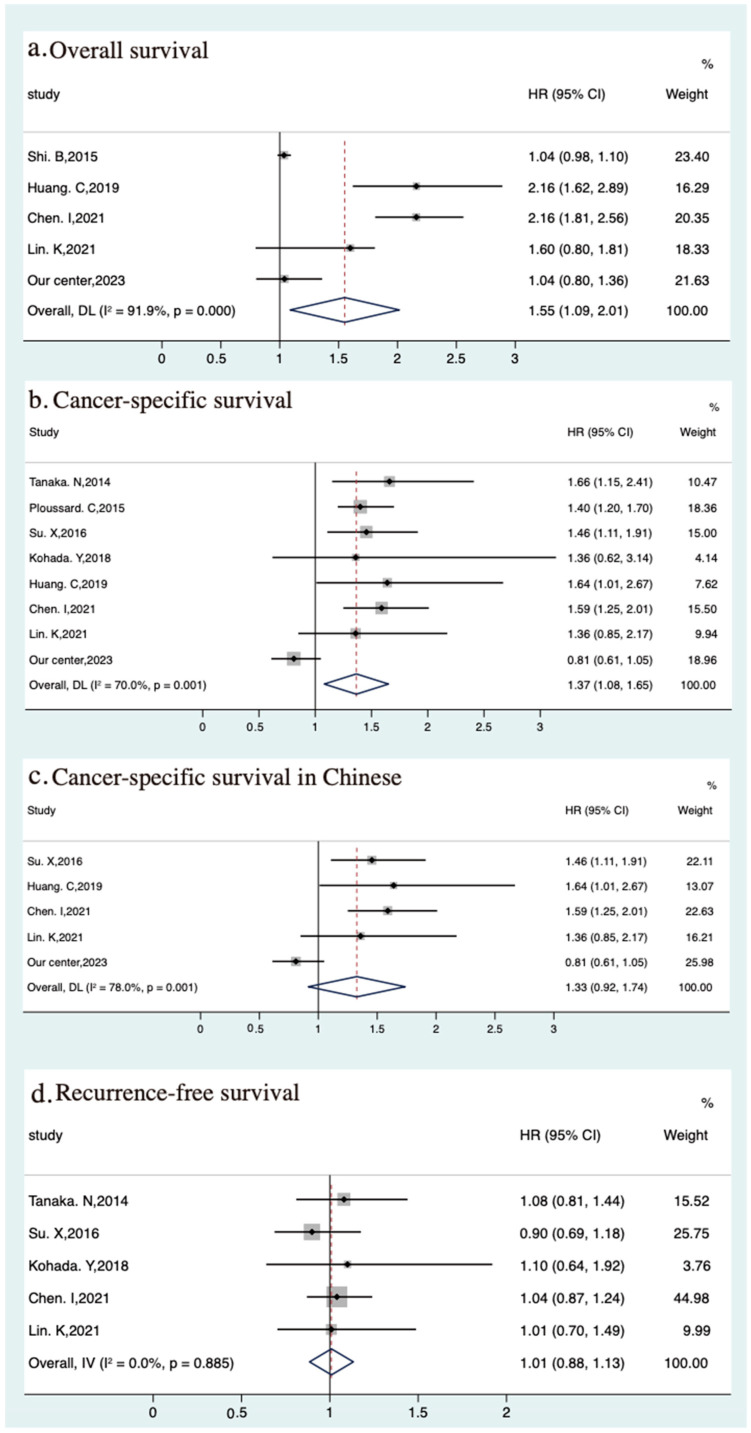
Meta-analyses of oncological outcomes of the elders (>70) vs. youngers: (**a**) overall survival [24,38,44,45], (**b**) cancer-specific survival [20,21,31,36,38,44,45], (**c**) cancer-specific survival in Chinese [31,38,44,45], (**d**) recurrence-free survival [20,31,36,44,45].

Data on the association between the age of 70 and OS and CSS were available from 5 and 10, respectively. For OS, one study conducted by Kuo et al. was excluded because of the similar publication year and institution, and the same region. In our center, age greater than 70 was not a significantly adverse factor for OS for UTUC patients after RNU (HR 1.04, 95% CI 0.80–1.36), which was also used for pooled analysis. High heterogeneity (*p* < 0.001, I^2^ = 91.9%) was observed among the five studies. Therefore, the pooled effect value of OS (pooled HR 1.55, 95% CI 1.29–2.01) was calculated using a random-effects model (Figure 3a).

Among the ten studies on CSS, two studies with similar regions and institutions and one study with information on cancer-specific mortality rather than CSS were excluded. Then, data from the remaining studies and data from our single-center case series (HR, 0.81, 95% CI 0.61–1.05) were used for meta-analysis, and the results indicated a significant association between age greater than 70 and poor CSS (pooled HR, 1.37, 95% CI 1.08–1.65). Significant heterogeneity was observed in the primary analysis (*p* = 0.001; I^2^ = 70.0%) (Figure 3b). Interestingly, to explore the influence of region on the association of age with CSS, we extracted five studies from China, and the pooled HR estimate for CSS was 1.33 (95% CI, 0.92–1.74), indicating no clear correlation between age and CSS; there was high heterogeneity among these studies (*p* = 0.001; I^2^ = 78.0%) (Figure 3c).

Due to the number restriction of the included studies, we utilized Egger regression to evaluate publication bias, and no obvious publication bias was observed in those studies (*p* = 0.193 for OS, *p* = 0.900 for CSS, *p* = 0.861 for sub-CSS).

## 4. Discussion

Population aging has posed a substantial challenge for the management of UTUC around the world. The increase in UTUC patients, characterized by localized lesions on first diagnosis, has been attributed to the strengthening of health awareness and widespread use of CT [1]. In addition, advances in minimally invasive therapies (especially in laparoscopic and robotic technology) have assuaged concerns about conventional surgery, offered advantages associated with less blood loss, decreased pain, and faster operation time, and created an opportunity to perform surgery on elderly patients [52,53]. However, the RNU for the aged UTUC patients is still controversial.

In the present study, we conducted a retrospective analysis to compare the survival outcomes (CSS) between octogenarians and younger patients and placed our findings in an international context by systematically reviewing the relevant literature. In our center, octogenarians with UTUC who underwent RNU showed a comparable outcome to younger UTUC patients. Further review of previous studies and meta-analyses indicated that advanced age (>70 years) tended to be related to poor OS and CSS. However, there was no significant association between advanced age and CSS in China.

Our results are in concordance with several single- and multi-institutional studies in which a negative association between the advanced age and prognosis of UTUC patients after RNU has been found [47,54]. In contrast, several previous studies have shown that age was an independent predictor of long-term survival for UTUC patients, and advanced age was found to be associated with worse outcomes [55,56]. However, few studies have been conducted on the topic of patient age, and our systematic review only found five studies on this topic. In 2009, a multicenter study of 1453 patients after RNU found that being older at the time of RNU was associated with inferior CSS, which was also observed by Stanley et al. in 2011 even after adjusting for tumor stage, grade, and treatment [12,56]. In contrast, the latest publication, studied by Koterazawa et al., retrospectively included 283 UTUC patients after laparoscopic RNU and demonstrated that no significant difference in the incidence of postoperative complications and 5-year OS, CSS, and RFS was observed between octogenarians and younger patients [51].

There might be several sources of the between-study heterogeneity observed herein. First, the majority of the previous studies only investigated chronological age, and few studies have considered anesthetic risk, biological age, comorbidities, and other potential factors. In 2018, biological age, evaluated by the categories of hemoglobin and white blood cells, was reported to show a stronger relationship with outcomes than chronological age in UTUC patients, especially elderly patients [57]. However, clinically, less aggressive, and nonstandard treatment regimens were recommended to patients based on their advancing age, and a patient’s chronological age alone may prevent them from receiving the best care for their disease. According to the American Society of Clinical Oncology guidelines, geriatric assessment (GA) should be used as intended to guide treatment decisions for elderly patients (≥65 years old) with cancer to identify vulnerabilities [58]. A recent study investigated the effects of age, ASA grading, and the Charlson Comorbidity Index (CCI) on UTUC outcomes and suggested that RNU should be a promising choice even among elderly patients who are eligible after GA [59]. However, Phaibulvatanapong et al. reported that CCI might not be suitable for assessing the comorbidity of cancer patients because cancer is one of the grading factors, which could result in patients with metastatic disease receiving an unjustified high score [60]. More future studies are needed to further explore and evaluate this in clinical practice.

In addition, the basic characteristics, such as race and region, may also cause selection bias. For UTUC, men in Europe and America typically have a 1.5–2.5 times higher risk than women, while in Asia, women have a 1.3 times higher risk than males [61,62,63]. Furthermore, being male was found to be associated with adverse outcomes in China rather than America, and the black race was found to be relevant to adverse outcomes in America [55,64]. This may partly explain the result of the pooled analysis that advanced age was significantly related to poor CSS, but the relationship disappeared when the region was restricted to China. However, in the above analysis, we failed to take gender and race into consideration. Sex in our center did not appear to be an independent predictor of CSS (HR 1.07; 95% CI 0.82, 1.40; *p* = 0.612), which was in line with most earlier studies. Interestingly, Kobayashi et al. evaluated the impact of gender-adjusted age on prognosis in upper tract urothelial carcinoma patients following RNU and demonstrated that older age was significantly associated with CSS independently of pathological features in male patients but not in female patients [28]. Moreover, in 2013, Liu et al. reviewed the data from 285 men and 136 women treated with RNU for UTUC, and the results showed that the impact of gender on UTUC outcomes is age-specific [65]. Females aged ≥59 years had worse outcomes than their male counterparts, while women <42 years and 42–58 years had better outcomes than men. Sex hormones and their receptors were thought to be the cause of gender differences in outcomes. Therefore, further research is needed to confirm whether sex- or age-specific sex has an effect on UTUC outcomes and to elucidate the molecular mechanism.

According to the EAU guidelines, the curative therapy for UTUC remains RNU, while nephron-sparing surgery or several novel technologies, such as ablation, are a choice for low-risk UTUC and patients with solitary kidney or severe renal impairment [2]. Older patients should not be denied potentially curative surgery based solely on their age; instead, we should make every effort to assess the overall condition of older patients. Recently, other studies have also demonstrated that indicators that reflect preoperative inflammation or nutritional status, such as the neutrophil-to-lymphocyte ratio, monocyte-to-lymphocyte ratio, and platelet-to-lymphocyte ratio, are associated with worse outcomes in patients with carcinoma [66,67]. Therefore, an authoritative evaluation system or nomogram aimed at identifying a special population of elderly patients who can benefit from RNU is urgently needed to guide clinical practice.

Notably, the strength of our study was that we provided a comprehensive reference about the relationship between the chronological age of UTUC patients at the time of RNU and survival outcomes and suggested that advanced age may not be an absolute contraindication for RNU by combining our single-center findings with a systematic review as well as a meta-analysis. Nonetheless, our study had some limitations. For our cases, the small size of octogenarians and the absence of specific comorbidities, due to the retrospective setting, are the most notable. Moreover, studies that focused on the influence of age were scarce, and age tended to be a continuous variable in most studies. The cut-off value for age in the pooled analysis was 70, which was limited by the number of included studies. However, it is relatively common for patients with newly diagnosed UTUC to be older than 70 years, whereas we were interested in the age of 80 years, which limits the external application of the conclusions of our study. Admittedly, in this single-center case series, we only chose the ASA score to reflect the general condition of patients from the perspective of tolerance to anesthesia. However, some novel indicators, which were determined by preoperative status and previously reported to influence the prognosis of UTUC patients, were not included in our analysis. These indicators included body composition based on computed tomography scans (e.g., iliopsoas muscle area, visceral fat density, intermuscular fat area, muscle attenuation, etc.), preoperative nutrition and inflammatory status (e.g., Naples prognostic score, controlling nutritional status score, prognostic nutritional index, etc.), and other indexes (e.g., CCI, modified frailty index, etc.). We are conducting a prospective, multicenter study with improved baseline balance and more complete basal characteristics to address these limitations.

## 5. Conclusions

Our single-center case series and the systematic review indicated that advanced age may not be an absolute contraindication for RNU in UTUC patients. RNU can be a safe and effective choice for UTUC patients of advanced age after a comprehensive presurgical evaluation.

## Figures and Tables

**Table 1:** The clinical and pathological characteristics of patients.

	Total (*n* = 588)	The Young Group (*n* = 531)	The Octogenarian Group (*n* = 57)	*p* Value
Age (IQR)	68.0 (60.0–75.0)	67.0 (59.0–73.0)	83.0 (81.0–85.0)	
Gender, *n* (%)				0.238
Men	322 (54.8)	295 (55.6)	27 (47.4)	
Women	266 (45.2)	236 (44.4)	30 (52.6)	
Body mass index (IQR)	22.7 (20.7–25.2)	22.8 (20.8–25.4)	21.1 (19.0–24.0)	0.042
Albumin (IQR)	40.2 (37.0–43.0)	40.6 (37.2–43.0)	38.9 (35.0–41.4)	0.019
ASA score, *n* (%)				<0.001
ASA ≤ 2	323 (54.93)	317 (59.70)	6 (10.53)	
ASA ≥ 3	265(45.07)	214 (40.30)	51 (89.47)	
Anemia, *n* (%)				0.119
Yes	242 (41.2)	213 (40.2)	29 (50.9)	
No	345 (58.8)	317 (59.8)	28 (49.1)	
Tumor location, *n* (%)				0.834
Renal pelvis	386 (65.8)	348 (65.7)	38 (66.7)	
Ureter	136 (23.2)	122 (23.0)	14 (24.6)	
Both	65 (11.1)	60 (11.3)	5 (8.8)	
Tumor stage, *n* (%)				0.023
Tis/Ta/T1	177 (30.3)	152 (28.8)	25 (43.9)	
T2	116 (19.8)	105 (19.9)	11 (19.3)	
T3	204 (34.9)	185 (35.0)	19 (33.3)	
T4	88 (15.0)	86 (16.3)	2 (3.5)	
Tumor grade, *n* (%)				0.638
High	435 (74.6)	391 (74.3)	44 (77.2)	
Low	148 (25.4)	135 (25.7)	13 (22.8)	
Tumor size, *n* (%)				0.425
≥3	387 (66.2)	352 (66.7)	35 (61.4)	
<3	198 (33.8)	176 (33.3)	22 (38.6)	
Lymphovascular invasion, *n* (%)				0.712
Yes	92 (15.7)	84 (15.9)	8 (14.0)	
No	493 (84.3)	444 (84.1)	49 (86.0)	
Lymph node status, *n* (%)				0.102
pN0/x	530 (90.4)	475 (89.8)	55 (96.5)	
pN+	56 (9.6)	54 (10.2)	2 (3.5)	
Multifocal, *n* (%)				0.821
Yes	98 (16.8)	88 (16.7)	10 (17.9)	
No	486 (83.2)	440 (83.3)	46 (82.1)	
Tumor necrosis, *n* (%)				0.849
Yes	45 (7.7)	41 (7.7)	4 (7.0)	
No	543 (92.3)	490 (92.3)	53 (93.0)	
Surgery margin, *n* (%)				0.163
Positive	49 (8.4)	47 (8.9)	2 (3.5)	
Negative	536 (91.6)	481 (91.1)	55 (96.5)	
Tumor architecture, *n* (%)				0.229
Papillary	319 (54.9)	292 (55.7)	27 (47.4)	
Sessile	262 (45.1)	232 (44.3)	30 (52.6)	
Surgery approach, *n* (%)				0.359
Open	345 (58.7)	313 (58.9)	32 (56.1)	
Laparoscopic	243 (41.3)	218 (41.1)	25 (43.9)	
Adjuvant systemic chemotherapy, *n* (%)				0.548
Yes, after RNU	47 (7.99)	43 (8.10)	4 (7.02)	
Yes, after progression	41 (6.97)	38 (7.15)	3 (5.26)	
No	500 (85.04)	450 (84.75)	50 (87.72)	

Abbreviations: IQR = Interquartile Range, ASA = American Society of Anesthesiologists, RNU = Radical nephroureterectomy.

**Table 2:** Univariate and multivariate analyses of cancer-specific survival.

Variables	Cancer-Specific Survival			
	Univariable Analyses		Multivariable Analyses	
	HR (95% CI)	*p* Value	HR (95% CI)	*p* Value
Body mass index	1.05 (0.97, 1.14)	0.200		
Anemia	1.89 (1.45, 2.46)	<0.001	1.59 (0.95, 1.98)	0.089
Albumin	0.97 (0.95, 0.99)	0.018	1.00 (0.97, 1.02)	0.752
Tumor necrosis	1.41 (0.89, 2.23)	0.145		
Multifocal	0.95 (0.66, 1.36)	0.776		
Lymphovascular invasion	2.53 (1.86, 3.44)	<0.001	1.27 (0.90, 1.80)	0.173
Age (Octogenarian vs. younger)	0.80 (0.50, 1.30)	0.376	1.08 (0.48, 2.40)	0.853
Gender (male vs. female)	1.07 (0.82, 1.40)	0.612		
ASA score (ASA ≥ 3 vs. ASA ≤ 2)	1.22 (0.80, 1.88)	0.352		
Tumor site (ureter vs. renal pelvis)	1.38 (1.02, 1.88)	0.039	1.54 (1.12, 2.14)	0.009
(both vs. renal pelvis)	1.44 (0.97, 2.13)	0.069	1.82 (1.21, 2.74)	0.004
Tumor stage (≥T3 vs. <T3)	3.74 (2.78, 5.04)	<0.001	2.39 (1.70, 3.35)	0.003
Tumor grade (high vs. low)	3.00 (2.02, 4.46)	<0.001	1.86 (1.22, 2.84)	0.004
Tumor size (≥3 vs. <3)	2.00 (1.47, 2.73)	<0.001	1.73 (1.24, 2.40)	0.001
Tumor architecture (papillary vs. sessile)	1.54 (1.17, 2.03)	0.002	0.99 (0.74, 1.34)	0.967
Lymph node status (pN+ vs. pN0/x)	3.84 (2.75, 5.36)	<0.001	2.10 (1.46, 3.02)	<0.001
Surgery approach (open vs. laparoscopic)	1.12 (0.87, 1.34)	0.102		
Surgery margin (positive vs. negative)	2.31 (1.56, 3.42)	<0.001	1.20 (0.92, 1.83)	0.392
Adjuvant systemic chemotherapy (yes vs. no)	0.78 (0.43, 1.03)	0.069	0.89 (0.59, 1.21)	0.198

Abbreviations: HR = Hazard Ratios, CI = Confidential Interval, ASA = American Society of Anesthesiologists.

**Table 3:** The characteristics of the included studies.

Study	Year	Country	Period	No. of Patients	Median Age	Age Group	Number of Age Group	Follow-Up	RNU Surgery	OS HR, 95% CI	CSS HR, 95% CI	RFS HR, 95% CI
SuleymanA. [10]	2006	Turkey	1993–2003	24	61.0	<60 >60	NA	34.8	NA	NS	-	-
Li C. [11]	2008	China	1990–2005	260	65	<65 >65	130 130	52.0	Open (80.4%) Lapa (19.6%)		<65 >65: 0.61 (0.32–1.18)	>65: 1.16 (0.69–1.94) (IVRFS)
Shariat S. [12]	2009	Multi-	1987–2007	1453	69.7	<50 50–59.9 60–69.9 70–79.9 >80	85 229 416 523 200	48.0	NA	<50 50–59: 0.92 60–69: 1.52 70–79: 1.78 >80: 2.51	<50 50–59: 0.81, NS 60–69: 1.30, NS 70–79: 1.09, NS >80: 1.63, S	-
Chromechi T. [13]	2011	Multi-	1987–2009	1169	69.0	<50 50–59.9 60–69.9 70–79.9 >80	66 185 367 419 132	37.0	NA	<50 50–59: 1.94 (0.90–4.18) 60–69: 3.38 (1.65–6.94) 70–79: 4.96 (2.43–10.12) >80: 7.79 (3.70–16.42)	<50 50–59: 1.68 (0.73–3.85) 60–69: 2.46 (1.13–5.36) 70–79: 2.71 (1.25–5.89) >80: 4.21(1.84–9.64)	<50 50–59: 1.48 (0.75–2.89) 60–69: 1.73 (0.92–3.25) 70–79: 1.99 (1.06–3.72) >80: 2.72 (1.37–5.39)
Milojevic B. [14]	2011	Serbia	1999–2009	133	66.6	<60 >60	NA	35.0	NA	-	<60 >60: 1.16 (0.52–2.73)	-
Metcalfe M. [15]	2012	Canada	1990–2010	1029	69.7	<65 >65	NA	26.4	Open (39.1%) Lapa (60.0%)	<65 >65: 1.05 (1.03–1.06)	<65 >65: 1.03 (1.01–1.06)	<65 >65: 1.02 (1.01–1.04)
Lim S. [16]	2013	Korea	2007–2010	32	66.5	<60 >60	NA	45.5	RALRNU	-	NS	NS
Li W. [17]	2013	China	1990–2007	127	66.3	<66 >66	58 69	40.0	Open (72.4%) Lapa (27.6%)	-	NS	NS
Obata J. [18]	2013	Japan	1993–2009	183	NA	<70 >70	76 107	39.0	Open (66.0%) Lapa (34.0%)	-	NS	NS
Luo H. [19]	2014	China	2005–2010	234	66.9	<70 >70	NA	40.7	NA	-	<70 >70: 5.01 (1.44–17.45)	-
Tanaka N. [20]	2014	Japan	1993–2011	665	70	<70 >70	333 332	28.0.0	NA	-	<70 >70: 1.66 (1.15–2.41)	<70 >70: 1.08 (0.81–1.44)
Ploussard G. [21]	2015	Multi-	1989–2012	3544	70	<70 >70	1772 1772	32.7	Open (74.0%) Lapa (26.0%)	-	<70 >70: 1.4 (1.2–1.7)	>70: 1.3 (1.1–1.5) (IVRFS)
Lee H. [22]	2015	China	2004–2010	250	NA	<68 >68	111 139	41.0	Open (66.4%) Lapa (33.6%)	-	<68 >68: 0.94 (0.50–1.76)	68: 1.12 (0.72–1.76) (IVRFS)
Morizane S. [23]	2015	Japan	2000–2012	345	74	<75 >75	182 163	39.9	Open (70.7%) Lapa (29.3%)	-	NA	-
Shi B. [24]	2015	China	2002–2010	176	69	<70 >70	84 92	36.0	Open (46.0%) Lapa (54.0%)	<70 >70: 1.036 (0.981–1.095)	<70 >70: 0.996 (0.969–1.023)	
Tanaka N. [25]	2015	Japan	1995–2011	394	70	<70 >70	205 189	30.0	NA	-	<70 >70: 1.56 (1.01–2.41)	-
Zhang X. [26]	2015	China	1990–2011	100	60.3	<60 >60	49 51	45.8	NA	<60 >60: 1.71 (0.88–3.29)		<60 >60: 1.80 (0.93–3.47)
Cheng Y. [27]	2015	China	2005–2010	195	68	<68 >68	85 110	36.0	NA	<68 >68: 1.269 (0.738–2.182)	<68 >68: 0.919 (0.486–1.741)	-
Kobayashi H. [28]	2016	Japan	1990–2011	839	70.4	<60 60–69.9 70–79.9 >80	156 245 315 123	34.0	Open (NA%) Lapa (NA%)	-	<60 60–69: 1.47 (0.91–2.37) 70–79: 1.62 (1.02–2.58) >80: 2.64 (1.56–4.48)	NS
Liang C. [29]	2016	China	2001–2014	172	70	<70 >70	86 86	44.0	Open (83%) Lapa (17%)	-	NS	-
Yan S. [30]	2016	China	2002–2012	795	NA	<67 >67	389 406	32.0	Open (74.0%) Lapa (26.0%)	<67 >67: 0.892 (0.725–1.097)	<67 >67: 1.196 (0.958–1.494)	<67 >67: 0.744 (0.59–0.937)
Su X. [31]	2016	China	1999–2011	687	NA	<70 >70	409 278	65.0	NA	-	<70 >70: 1.455 (1.107–1.912)	<70 >70: 0.899 (0.687–1.176)
Cao Z. [32]	2017	China	2001–2011	656	68	<60 >60	160 496	46.0	NA	-	<60 >60: 1.303 (0.845–2.010)	>60: 0.777(0.535–1.129) (IVRFS)
Daimon T. [33]	2017	Japan	1984–2007	165	66.4	<70 >70	94 71	62.4	Open (79.4%) Lapa (20.6%)	-	NS	NS
Huang J. [34]	2017	China	2002–2013	481	65.8	<65 >65	207 274	40.0	Open (66.1%) Lapa (33.9%)	<65 >65: 2.38 (1.62–3.50)	<65 >65: 2.00 (1.31–3.03)	-
Kim J. [35]	2017	Korea	1991–2012	452	64.0	<60 >60	148 304	67.8	Open (73.5%) Lapa (26.5%)	<60 >60: 2.619 (1.720–3.986)	<60 >60: 2.018 (1.265–3.219)	-
Kohada Y. [36]	2018	Japan	1999–2016	148	71	<70 >70	72 76	35.5	Open (NA%) Lapa (NA%)	-	<70 >70: 1.36 (0.62–3.14)	<70 >70: 1.1 (0.64–1.92)
Zeng S. [37]	2019	China	2008–2018	445	66.3	<65 >65	NA	33.5	NA	-	<65 >65: 1.46 (0.96–2.24)	-
Huang C. [38]	2019	China	2004–2013	506	67	<70 >70	295 211	82.8	NA	<70 >70: 2.16 (1.62–2.89)	<70 >70: 1.64 (1.01–2.67)	-
Chung H. [39]	2019	Korea	2002–2016	186	68.8	<69 >69	96 90	38.9	NA	<69 >69: 1.37 (0.76–2.49)	<69 >69: 1.16 (0.58–2.30)	<69 >69: 0.98 (0.66–1.47)
Kuroda K. [40]	2019	Japan	1999–2017	187	71	<71 >71	93 94	49.2	Open (55.6%) Lapa (44.4%)	-	NS	NS
Chen X. [41]	2020	China	2008–2018	232	65	<65 >65	116 116	39.0	Open (38.4%) Lapa (61.6%)	<65 >65: 1.29 (0.85–1.97)	<65 >65: 1.27 (0.81–2.01)	<65 >65: 1.39 (0.95–2.02)
Liu W. [42]	2020	China	2012–2019	315	67	<65 >65	121 194	32.0	NA	<65 >65: 2.09 (1.11–3.93)	<65 >65: 2.05 (1.08–3.90)	<65 >65: 2.45 (1.32–4.53)
Azawi N. [43]	2020	Denmark	2004–2017	1384	70	<60 60–69.9 70–79.9 >80	214 499 539 132	54.0	Open (23%) Lapa (77%)	<60 60–69: 1.74 (1.16–2.61) 70–79: 1.94 (1.31–2.92) >80: 2.37 (1.43–3.95)	-	<60 60–69: 0.84 (0.59–1.18) 70–79: 0.94 (0.67–1.31) >80: 0.59 (0.35–1.01)
Chen I. [44]	2021	China	1988–2021	1808	69	<70 >70	910 898	41.8	Open (21.1%) Non-open (78.9)	<70 >70: 2.16 (1.81–2.56)	<70 >70: 1.59 (1.25–2.01)	<70 >70: 1.04 (0.87–1.24)
Lin K. [45]	2021	China	2008–2019	521	69.1	<70 >70	249 272	49.1	Lapa (100%)	<70 >70: 1.599 (0.795–1.808)	<70 >70: 1.359 (0.849–2.174)	<70 >70: 1.009 (0.703–1.488)
Milojevic B. [46]	2021	Serbia	2000–2018	342	66.6	<60 >60	NA	32.5	NA	-	<60 >60: 1.52 (0.79–2.90)	<60 >60: 0.83 (0.46–1.49)
Yamada Y. [47]	2022	Japan	1990–2015	441	69	≤64 65–79 ≥80	135 254 52	NA	Open (56.0%) Lapa (44.0%)	≤64 65–79: 1.3 (0.8–2.0) ≥80: 3.0 (1.6–5.8)	≤64 65–79: 0.8 (0.5–1.4) ≥80: 1.5 (0.7–3.2)	≤64 65–79: 0.8 (0.5–1.2) ≥80: 1.1 (0.6–2.1)
Kuo C. [48]	2022	China	2002–2019	322	69	<70 >70	NA	62.1 30.3	HALNU (43.8%) Lapa (56.2%)	<70 >70: 4.15 (1.85–9.30)	<70 >70: 20.31 (2.22–185.77)	<70 >70: 1.561 (0.97–2.51)
Gao J. [49]	2022	China	2010–2020	401	67	<67 >67	200 201	44.7	Open (NA%) Lapa (NA%)	<67 >67: 1.41 (0.97–2.05)	<67 >67: 1.17 (0.77–1.77)	<67 >67: 0.96 (0.68–1.36)
Ke H. [50]	2022	China	2000–2015	409	69.5	<65 >65	182 227	NA	Open (71.9%) Lapa (28.1%)	<65 >65: 0.97 (0.65–1.45)	<65 >65: 0.79 (0.51–1.22)	-
Koterazawa S. [51]	2023	Japan	2002–2020	283	74.5	<80 >80	213 70	36.3 18.0	Lapa (100%)	-	<80 >80: 0.77 (0.38–1.51)	-

Abbreviations: HR Hazard Ratios, CI Confidential Interval, RNU = Radical Nephroureterectomy, NA = Not Available; NS = Non-significant; OS = Overall Survival; CSS = Cancer-Specific Survival; RFS = Recurrence-Free Survival; RALRNU = Robot-Assisted Laparoscopic Nephroureterectomy; HALNU = Hand-Assisted Laparoscopic Nephroureterectomy.

## Data Availability

The data that support the findings of this study are available on request from the corresponding author, upon reasonable request.

## Supplementary Materials

The following supporting information can be downloaded at: https://www.mdpi.com/article/10.3390/jcm12237273/s1. Table S1: The PRISMA checklist of the present study. Table S2: Newcastle-Ottawa Scale for assessing the quality of included studies. References [1,2,3,4,5,6,7,8,9,10,11,12,13,14,15,16,17,18,19,20,21,22,23,28,47,51,52,53,54,55,56,57,58,59,60,61,62,63,64,65,66,67,68] are cited in the Supplementary Materials.
