# Supplementary material for: Advanced Age May Not Be an Absolute Contraindication for Radical Nephroureterectomy in Patients with Upper Tract Urothelial Carcinoma: A Single-Center Case Series and a Systematic Review with Meta-Analyses
Source: J Clin Med. 2023 Nov 24;12(23):7273. doi: 10.3390/jcm12237273 (PMC10707095; doi:10.3390/jcm12237273)
Supplement: Supplementary file 1 [file jcm-12-07273-s001.zip › jcm-2653091-supplementary.pdf]

Supplementary Table S1. The PRISMA checklist of the present study.

Section and Topic	Item #	Checklist item	Location where item is reported
**TITLE**			
Title	1	Identify the report as a systematic review.	Page 1<br>Line 1-4
**ABSTRACT**			
Abstract	2	See the PRISMA 2020 for Abstracts checklist.	Page 2<br>Line 17-36
**INTRODUCTION**			
Rationale	3	Describe the rationale for the review in the context of existing knowledge.	Page 3<br>Line 40-53
Objectives	4	Provide an explicit statement of the objective(s) or question(s) the review addresses.	Page 3<br>Line 54-58
**METHODS**			
Eligibility criteria	5	Specify the inclusion and exclusion criteria for the review and how studies were grouped for the syntheses.	Page 6<br>Line 114-120
Information sources	6	Specify all databases, registers, websites, organisations, reference lists and other sources searched or consulted to identify studies. Specify the date when each source was last searched or consulted.	Page 6<br>Line 109-113
Search strategy	7	Present the full search strategies for all databases, registers and websites, including any filters and limits used.	Page 6<br>Line 109-113
Selection process	8	Specify the methods used to decide whether a study met the inclusion criteria of the review, including how many reviewers screened each record and each report retrieved, whether they worked independently, and if applicable, details of automation tools used in the process.	Page 6<br>Line 122-123
Data collection process	9	Specify the methods used to collect data from reports, including how many reviewers collected data from each report, whether they worked independently, any processes for obtaining or confirming data from study investigators, and if applicable, details of automation tools used in the process.	Page 6<br>Line 122-126
Data items	10a	List and define all outcomes for which data were sought. Specify whether all results that were compatible with each outcome domain in each study were sought (e.g. for all measures, time points, analyses), and if not, the methods used to decide which results to collect.	Page 6<br>Line 123-126
	10b	List and define all other variables for which data were sought (e.g. participant and intervention characteristics, funding sources). Describe any assumptions made about any missing or unclear information.	Page 6<br>Line 123-126
Study risk of bias assessment	11	Specify the methods used to assess risk of bias in the included studies, including details of the tool(s) used, how many reviewers assessed each study and whether they worked independently, and if applicable, details of automation tools used in the process.	Page 6-7<br>Line 127-130
Effect measures	12	Specify for each outcome the effect measure(s) (e.g. risk ratio, mean difference) used in the synthesis or presentation of results.	Page 6<br>Line 135
Synthesis methods	13a	Describe the processes used to decide which studies were eligible for each synthesis (e.g. tabulating the study intervention characteristics and comparing against the planned groups for each synthesis (item #5)).	Page 6<br>Line 132-134
	13b	Describe any methods required to prepare the data for presentation or synthesis, such as handling of missing summary statistics, or data conversions.	
	13c	Describe any methods used to tabulate or visually display results of individual studies and syntheses.	Page 7<br>Line 132
	13d	Describe any methods used to synthesize results and provide a rationale for the choice(s). If meta-analysis was performed, describe the model(s), method(s) to identify the presence and extent of statistical heterogeneity, and software package(s) used.	Page 7<br>Line 132-138
	13e	Describe any methods used to explore possible causes of heterogeneity among study results (e.g. subgroup analysis, meta-regression).	Page 7<br>Line 133-134
	13f	Describe any sensitivity analyses conducted to assess robustness of the synthesized results.	Page 7<br>Line 133-134
Reporting bias assessment	14	Describe any methods used to assess risk of bias due to missing results in a synthesis (arising from reporting biases).	Page 7<br>Line 127-130
Certainty assessment	15	Describe any methods used to assess certainty (or confidence) in the body of evidence for an outcome.	Page 6<br>Line 127-130
**RESULTS**			
Study selection	16a	Describe the results of the search and selection process, from the number of records identified in the search to the number of studies included in the review, ideally using a flow diagram.	Page 8<br>Line 171-173
	16b	Cite studies that might appear to meet the inclusion criteria, but which were excluded, and explain why they were excluded.	Page 8

Section and Topic	Item #	Checklist item	Location where item is reported
			Line 171-173
Study characteristics	17	Cite each included study and present its characteristics.	Page 8<br>Line 173
Risk of bias in studies	18	Present assessments of risk of bias for each included study.	Page 9<br>Line 185-188
Results of individual studies	19	For all outcomes, present, for each study: (a) summary statistics for each group (where appropriate) and (b) an effect estimate and its precision (e.g. confidence/credible interval), ideally using structured tables or plots.	Page 8<br>Line 171-173
Results of syntheses	20a	For each synthesis, briefly summarise the characteristics and risk of bias among contributing studies.	Page 8-9<br>Line 190-211
	20b	Present results of all statistical syntheses conducted. If meta-analysis was done, present for each the summary estimate and its precision (e.g. confidence/credible interval) and measures of statistical heterogeneity. If comparing groups, describe the direction of the effect.	Page 8-9<br>Line 190-211
	20c	Present results of all investigations of possible causes of heterogeneity among study results.	Page 9<br>Line 205-208
	20d	Present results of all sensitivity analyses conducted to assess the robustness of the synthesized results.	Page 9<br>Line 205-208
Reporting biases	21	Present assessments of risk of bias due to missing results (arising from reporting biases) for each synthesis assessed.	Page 9<br>Line 185-188
Certainty of evidence	22	Present assessments of certainty (or confidence) in the body of evidence for each outcome assessed.	Page 10<br>Line 209-211
**DISCUSSION**			
Discussion	23a	Provide a general interpretation of the results in the context of other evidence.	Page 11<br>Line 221-227
	23b	Discuss any limitations of the evidence included in the review.	Page 14-15<br>Line 294-311
	23c	Discuss any limitations of the review processes used.	Page 14-15<br>Line 294-311
	23d	Discuss implications of the results for practice, policy, and future research.	Page 14<br>Line 290-294<br>Line 309-311
**OTHER INFORMATION**			
Registration and protocol	24a	Provide registration information for the review, including register name and registration number, or state that the review was not registered.	NA
	24b	Indicate where the review protocol can be accessed, or state that a protocol was not prepared.	NA
	24c	Describe and explain any amendments to information provided at registration or in the protocol.	NA
Support	25	Describe sources of financial or non-financial support for the review, and the role of the funders or sponsors in the review.	Page 16<br>Line 322-325
Competing interests	26	Declare any competing interests of review authors.	Page 16<br>Line 318
Availability of data, code and other materials	27	Report which of the following are publicly available and where they can be found: template data collection forms; data extracted from included studies; data used for all analyses; analytic code; any other materials used in the review.	Page 16<br>Line 327-328

From: Page MJ, McKenzie JE, Bossuyt PM, Boutron I, Hoffmann TC, Mulrow CD, et al. The PRISMA 2020 statement: an updated guideline for reporting systematic reviews. BMJ 2021;372:n71. doi: 10.1136/bmj.n71

For more information, visit: http://www.prisma-statement.org/

Supplementary Table S2. Newcastle-Ottawa Scale for assessing the quality of included studies.

Study	Selection				Comparability	Expodsure			
	Representativeness of the exposed cohort	Selection of the non-exposed cohort	Ascertainment of exposure	Demonstration that outcome of interest was not present at start of study	Comparability of cohorts on the basis of the design or analysis	Assessment of outcome	Was follow-up long enough for outcomes to occur	Adequacy of follow up of cohorts	Total scores
**Suleyman. A[1]**	★	★		★	★	★	★	★	7
**Li. C[2]**	★	★		★	★	★	★	★	7
**Shariat. S[3]**	★	★	★	★	★★	★	★	★	9
**Chromechi. T[4]**	★	★	★	★	★★	★	★	★	9
**Milojevic.B[5]**	★	★	★	★	★	★	★	★	8
**Metcalfe. M[6]**	★	★		★	★	★	★	★	7
**Lim. S[7]**	★	★		★	★	★	★	★	7
**Li. W[8]**	★	★	★	★	★	★	★	★	8
**Obata. J[9]**	★	★	★	★	★	★	★	★	8
**Luo. H[10]**	★	★		★	★	★	★	★	7
**Tanaka. N[11]**	★	★		★	★	★		★	6
**Ploussard. G[12]**	★	★	★	★	★★	★	★	★	9
**Lee. H[13]**	★	★	★	★	★	★	★	★	8
**Morizane. S[14]**	★	★	★	★	★	★	★	★	8
**Shi. B[15]**	★	★	★	★	★	★	★	★	8
**Tanaka.N[16]**	★	★		★	★	★		★	6
**Zhang. X[17]**	★	★	★	★	★	★	★	★	8
**Cheng. Y[18]**	★	★	★	★	★	★	★	★	8
**Kobayashi. H[19]**	★	★	★	★	★★	★	★	★	9
**Liang. C[20]**	★	★		★	★	★	★	★	7
**Yan. S[21]**	★	★	★	★	★	★	★	★	8
**Su. X[22]**	★	★	★	★	★	★	★	★	8
**Cao. Z[23]**	★	★	★	★	★	★	★	★	8
**Daimon. T[24]**	★	★	★	★	★	★	★	★	8
**Huang, J[25]**	★	★	★	★	★	★	★	★	8
**Kim.J[26]**	★	★	★	★	★	★	★	★	8
**Kohada. Y[27]**	★	★		★	★	★	★	★	7
**Zeng. S[28]**	★	★		★	★	★		★	6
**Huang. C[29]**	★	★	★	★	★★	★	★	★	9
**Chung. H[30]**	★	★	★	★	★	★	★	★	8
**Kuroda. K[31]**	★	★		★	★	★	★	★	7
**Chen. X[32]**	★	★		★	★	★	★	★	7
**Liu. W[33]**	★	★	★	★	★	★		★	7
**Azawi. N[34]**	★	★	★	★	★★	★	★	★	9
**Chen. I[35]**	★	★	★	★	★★	★	★	★	9
**Lin. K[36]**	★	★	★	★	★	★	★	★	8
**Milojevic. B[37]**	★	★		★	★	★	★	★	7
**Yamada. Y[38]**	★	★	★	★	★	★	★	★	8
**Kuo. C[39]**	★	★		★	★★	★	★	★	8
**Gao. J[40]**	★	★		★	★★	★	★	★	8
**Ke. H[41]**	★	★	★	★	★★	★	★	★	9
**Koterazawa. S[42]**	★	★	★	★	★★	★		★	8

1. Ataus S, Onal B, Tunc B, Erozenci A, Cekmen A, Kural AR, et al. Factors affecting the survival of patients treated by standard nephroureterectomy for transitional cell carcinoma of the upper urinary tract. Int Urol Nephrol. 2006;38(1):9-13. doi: 10.1007/s11255-005-3151-3. PubMed PMID: 16502046.

2. Li CC, Chang TH, Wu WJ, Ke HL, Huang SP, Tsai PC, et al. Significant predictive factors for prognosis of primary upper urinary tract cancer after radical nephroureterectomy in Taiwanese patients. Eur Urol. 2008;54(5):1127-34. Epub 20080128. doi: 10.1016/j.eururo.2008.01.054. PubMed PMID: 18243511.

3. Shariat SF, Godoy G, Lotan Y, Droller M, Karakiewicz PI, Raman JD, et al. Advanced patient age is associated with inferior cancer-specific survival after radical nephroureterectomy. BJU Int. 2010;105(12):1672-7. Epub 20091113. doi: 10.1111/j.1464-410X.2009.09072.x. PubMed PMID: 19912201.

4. Chromecki TF, Ehdaie B, Novara G, Pummer K, Zigeuner R, Seitz C, et al. Chronological age is not an independent predictor of clinical outcomes after radical nephroureterectomy. World J Urol. 2011;29(4):473-80. Epub 20110417. doi: 10.1007/s00345-011-0677-0. PubMed PMID: 21499902.

5. Milojevic B, Djokic M, Sipetic-Grujicic S, Milenkovic-Petronic D, Vuksanovic A, Bumbasirevic U, et al. Upper urinary tract transitional cell carcinoma: location is not correlated with prognosis. BJU Int. 2012;109(7):1037-42. Epub 20110825. doi: 10.1111/j.1464-410X.2011.10461.x. PubMed PMID: 21883837.

6. Metcalfe M, Kassouf W, Rendon R, Bell D, Izawa J, Chin J, et al. Regional differences in practice patterns and associated outcomes for upper tract urothelial carcinoma in Canada. Can Urol Assoc J. 2012;6(6):455-62. doi: 10.5489/cuaj.12146. PubMed PMID: 23282664; PubMed Central PMCID: PMC3526631.

7. Lim SK, Shin TY, Kim KH, Chung BH, Hong SJ, Choi YD, et al. Intermediate-term outcomes of robot-assisted laparoscopic nephroureterectomy in upper urinary tract urothelial carcinoma. Clin Genitourin Cancer. 2013;11(4):515-21. Epub 20130627. doi: 10.1016/j.clgc.2013.04.027. PubMed PMID: 23810441.

8. Li WM, Wu WJ, Li CC, Ke HL, Wei YC, Yeh HC, et al. The effect of tumor location on prognosis in patients with primary ureteral urothelial carcinoma. Urol Oncol. 2013;31(8):1670-5. Epub 20120609. doi: 10.1016/j.urolonc.2012.05.004. PubMed PMID: 22687568.

9. Obata J, Kikuchi E, Tanaka N, Matsumoto K, Hayakawa N, Ide H, et al. C-reactive protein: a biomarker of survival in patients with localized upper tract urothelial carcinoma treated with radical nephroureterectomy. Urol Oncol. 2013;31(8):1725-30. Epub 20121108. doi: 10.1016/j.urolonc.2012.05.008. PubMed PMID: 23141922.

10. Luo HL, Chen YT, Chuang YC, Cheng YT, Lee WC, Kang CH, et al. Subclassification of upper urinary tract urothelial carcinoma by the neutrophil-to-lymphocyte ratio (NLR) improves prediction of oncological outcome. BJU Int. 2014;113(5b):E144-9. Epub 20140314. doi: 10.1111/bju.12582. PubMed PMID: 24274691.

11. Tanaka N, Kikuchi E, Kanao K, Matsumoto K, Shirotake S, Miyazaki Y, et al. A multi-institutional validation of the prognostic value of the neutrophil-to-lymphocyte ratio for upper tract urothelial carcinoma treated with radical nephroureterectomy. Ann Surg Oncol. 2014;21(12):4041-8. Epub 20140610. doi: 10.1245/s10434-014-3830-3. PubMed PMID:

24912614.

12. Ploussard G, Xylinas E, Lotan Y, Novara G, Margulis V, Rouprêt M, et al. Conditional survival after radical nephroureterectomy for upper tract carcinoma. Eur Urol. 2015;67(4):803-12. Epub 20140819. doi: 10.1016/j.eururo.2014.08.003. PubMed PMID: 25145551.

13. Lee HY, Li CC, Huang CN, Ke HL, Li WM, Liang PI, et al. Prognostic significance of lymphovascular invasion in upper urinary tract urothelial carcinoma is influenced by tumor location. Ann Surg Oncol. 2015;22(4):1392-400. Epub 20140920. doi: 10.1245/s10434-014-4103-x. PubMed PMID: 25239005.

14. Morizane S, Yumioka T, Yamaguchi N, Masago T, Honda M, Sejima T, et al. Risk stratification model, including preoperative serum C-reactive protein and estimated glomerular filtration rate levels, in patients with upper urinary tract urothelial carcinoma undergoing radical nephroureterectomy. Int Urol Nephrol. 2015;47(8):1335-41. Epub 20150624. doi: 10.1007/s11255-015-1033-x. PubMed PMID: 26104379.

15. Shi B, Su B, Fang D, Tang Y, Xiong G, Guo Z, et al. High expression of KPNA2 defines poor prognosis in patients with upper tract urothelial carcinoma treated with radical nephroureterectomy. BMC Cancer. 2015;15:380. Epub 20150509. doi: 10.1186/s12885-015-1369-8. PubMed PMID: 25956057; PubMed Central PMCID: PMC4432830.

16. Tanaka N, Kikuchi E, Kanao K, Matsumoto K, Shirotake S, Miyazaki Y, et al. Impact of Combined Use of Blood-based Inflammatory Markers on Patients with Upper Tract Urothelial Carcinoma Following Radical Nephroureterectomy: Proposal of a Cumulative Marker Score as a Novel Predictive Tool for Prognosis. Eur Urol Focus. 2015;1(1):54-63. Epub 20150523. doi: 10.1016/j.euf.2015.02.001. PubMed PMID: 28723357.

17. Zhang XK, Zhang ZL, Yang P, Cai MY, Hu WM, Yun JP, et al. Tumor necrosis predicts poor clinical outcomes in patients with node-negative upper urinary tract urothelial carcinoma. Jpn J Clin Oncol. 2015;45(11):1069-75. Epub 20150909. doi: 10.1093/jjco/hyv127. PubMed PMID: 26355163.

18. Cheng YC, Huang CN, Wu WJ, Li CC, Ke HL, Li WM, et al. The Prognostic Significance of Inflammation-Associated Blood Cell Markers in Patients with Upper Tract Urothelial Carcinoma. Ann Surg Oncol. 2016;23(1):343-51. Epub 20150805. doi: 10.1245/s10434-015-4781-z. PubMed PMID: 26242371.

19. Kobayashi H, Kikuchi E, Tanaka N, Shirotake S, Miyazaki Y, Ide H, et al. Patient age was an independent predictor of cancer-specific survival in male patients with upper tract urothelial carcinoma treated by radical nephroureterectomy. Jpn J Clin Oncol. 2016;46(6):554-9. Epub 20160309. doi: 10.1093/jjco/hyw028. PubMed PMID: 26962241.

20. Liang C, Chi R, Huang L, Wang J, Liu H, Xu D, et al. Upper Tract Urothelial Carcinomas Accompanied by Previous or Synchronous Nonmuscle-Invasive Bladder Cancer and Preoperative Hydronephrosis Might Have Worse Oncologic Outcomes After Radical Nephroureterectomy. Clin Genitourin Cancer. 2016;14(5):e469-e77. Epub 20160221. doi: 10.1016/j.clgc.2016.02.008. PubMed PMID: 27021588.

21. Shibing Y, Liangren L, Qiang W, Hong L, Turun S, Junhao L, et al. Impact of tumour size on prognosis of upper urinary tract urothelial carcinoma after radical nephroureterectomy: a multi-institutional analysis of 795 cases. BJU Int. 2016;118(6):902-10. Epub 20160327. doi: 10.1111/bju.13463. PubMed PMID: 26935344.

22. Su X, Fang D, Li X, Xiong G, Zhang L, Hao H, et al. The Influence of Tumor Size on Oncologic Outcomes for Patients with Upper Tract Urothelial Carcinoma after Radical Nephroureterectomy. Biomed Res Int. 2016;2016:4368943. Epub 20161214. doi: 10.1155/2016/4368943. PubMed PMID: 28070508; PubMed Central PMCID: PMC5192298.

23. Cao ZP, Guan B, Zhao GZ, Fang D, Xiong GY, Li XS, et al. Validation of the Pretreatment Neutrophil-to-Lymphocyte Ratio as a Prognostic Factor in a Large Cohort of Chinese Patients with Upper Tract Urothelial Carcinoma. Chin Med J (Engl). 2017;130(17):2063-8. doi: 10.4103/0366-6999.213414. PubMed PMID: 28836549; PubMed Central PMCID: PMC5586174.

24. Daimon T, Kosaka T, Kikuchi E, Mikami S, Miyazaki Y, Hashimoto A, et al. Prognostic significance of erythrocyte protein band 4.1-like5 expression in upper urinary tract urothelial carcinoma. Urol Oncol. 2017;35(9):543.e17-.e24. Epub 20170505. doi: 10.1016/j.urolonc.2017.04.008. PubMed PMID: 28483476.

25. Huang J, Yuan Y, Wang Y, Zhang J, Kong W, Chen H, et al. Prognostic value of preoperative plasma fibrinogen level and platelet-to-lymphocyte ratio (F-PLR) in patients with localized upper tract urothelial carcinoma. Oncotarget. 2017;8(22):36761-71. doi: 10.18632/oncotarget.13611. PubMed PMID: 27901490; PubMed Central PMCID: PMC5482695.

26. Kim JK, Moon KC, Jeong CW, Kwak C, Kim HH, Ku JH. Variant histology as a significant predictor of survival after radical nephroureterectomy in patients with upper urinary tract urothelial carcinoma. Urol Oncol. 2017;35(7):458.e9-.e15. Epub 20170324. doi: 10.1016/j.urolonc.2017.02.010. PubMed PMID: 28347659.

27. Kohada Y, Hayashi T, Goto K, Kobatake K, Abdi H, Honda Y, et al. Preoperative risk classification using neutrophil-lymphocyte ratio and hydronephrosis for upper tract urothelial carcinoma. Jpn J Clin Oncol. 2018;48(9):841-50. doi: 10.1093/jjco/hyy084. PubMed PMID: 30085174.

28. Zeng S, Dai L, Yang J, Gao X, Yu X, Ren Q, et al. Development and external validation of a nomogram predicting prognosis of upper tract urothelial carcinoma after radical nephroureterectomy. Urol Oncol. 2019;37(4):290.e17-.e24. Epub 20190108. doi: 10.1016/j.urolonc.2018.12.027. PubMed PMID: 30630733.

29. Huang CC, Su YL, Luo HL, Chen YT, Sio TT, Hsu HC, et al. Gender Is a Significant Prognostic Factor for Upper Tract Urothelial Carcinoma: A Large Hospital-Based Cancer Registry Study in an Endemic Area. Front Oncol. 2019;9:157. Epub 20190321. doi: 10.3389/fonc.2019.00157. PubMed PMID: 30949449; PubMed Central PMCID: PMC6437032.

30. Chung HS, Hwang EC, Kim MS, Yu SH, Jung SI, Kang TW, et al. Effects of Variant Histology on the Oncologic Outcomes of Patients With Upper Urinary Tract Carcinoma After Radical Nephroureterectomy: A Propensity Score-Matched Analysis. Clin Genitourin Cancer. 2019;17(3):e394-e407. Epub 20190111. doi: 10.1016/j.clgc.2018.11.015. PubMed PMID: 30782419.

31. Kuroda K, Asakuma J, Horiguchi A, Kawaguchi M, Shinchi M, Masunaga A, et al. Chronic kidney disease and positive surgical margins as prognosticators for upper urinary tract urothelial carcinoma patients undergoing radical nephroureterectomy. Mol Clin Oncol. 2019;10(5):547-54. Epub 20190320. doi: 10.3892/mco.2019.1829. PubMed PMID: 30967949; PubMed Central PMCID: PMC6449878.
32. Chen X, Ji H, Wang J, Zhao G, Zheng B, Niu Z, et al. Prognostic Value of the Preoperative Plasma D-Dimer Levels in Patients with Upper Tract Urothelial Carcinoma in a Retrospective Cohort Study. Onco Targets Ther. 2020;13:5047-55. Epub 20200608. doi: 10.2147/ott.S254514. PubMed PMID: 32606727; PubMed Central PMCID: PMC7292253.
33. Liu W, Wang Z, Liu S, Yao Y, Liu Y, Zhang G. Preoperative positive voided urine cytology predicts poor clinical outcomes in patients with upper tract urothelial carcinoma undergoing nephroureterectomy. BMC Cancer. 2020;20(1):1113. Epub 20201116. doi: 10.1186/s12885-020-07623-5. PubMed PMID: 33198698; PubMed Central PMCID: PMC7670807.
34. Azawi NH, Næraa SH, Subhi Y, Vásquez JL, Norus T, Dahl C, et al. Oncological outcomes of radical nephroureterectomy for upper urinary tract urothelial neoplasia in Denmark. Scand J Urol. 2020;54(1):58-64. Epub 20200116. doi: 10.1080/21681805.2019.1710562. PubMed PMID: 31942812.
35. Chen IA, Chang CH, Huang CP, Wu WJ, Li CC, Chen CH, et al. Factors Predicting Oncological Outcomes of Radical Nephroureterectomy for Upper Tract Urothelial Carcinoma in Taiwan. Front Oncol. 2021;11:766576. Epub 20220113. doi: 10.3389/fonc.2021.766576. PubMed PMID: 35096575; PubMed Central PMCID: PMC8793058.
36. Lin KC, Jan HC, Hu CY, Ou YC, Kao YL, Yang WH, et al. Tumor Necrosis with Adjunction of Preoperative Monocyte-to-Lymphocyte Ratio as a New Risk Stratification Marker Can Independently Predict Poor Outcomes in Upper Tract Urothelial Carcinoma. J Clin Med. 2021;10(13). Epub 20210703. doi: 10.3390/jcm10132983. PubMed PMID: 34279467; PubMed Central PMCID: PMC8267944.
37. Milojevic B, Bumbasirevic U, Santric V, Kajmakovic B, Dragicevic D, Radisavcevic D, et al. Prognostic significance of tumor multifocality on outcomes in patients with upper tract urothelial carcinoma after radical nephroureterectomy: A cohort study. Curr Probl Cancer. 2021;45(6):100747. Epub 20210415. doi: 10.1016/j.currproblcancer.2021.100747. PubMed PMID: 33883080.
38. Yamada Y, Ikeda M, Hirayama T, Murakami Y, Koguchi D, Matsuda D, et al. Noninferior oncological outcomes in adults aged 80 years or older compared with younger patients who underwent radical nephroureterectomy for upper tract urothelial carcinoma. Asia Pac J Clin Oncol. 2022. Epub 20220731. doi: 10.1111/ajco.13835. PubMed PMID: 35909301.
39. Kuo CC, Chen GH, Chang CH, Huang CY, Chen CH, Li CC, et al. Surgical outcome predictor analysis following hand-assisted or pure laparoscopic transperitoneal nephroureterectomy using the Taiwan upper urinary tract urothelial carcinoma database. Front Surg. 2022;9:934355. Epub 20220901. doi: 10.3389/fsurg.2022.934355. PubMed PMID:

36117820; PubMed Central PMCID: PMC9475171.

40. Gao J, Liu J, Liu J, Lin S, Ding D. Survival and risk factors among upper tract urothelial carcinoma patients after radical nephroureterectomy in Northeast China. Front Oncol. 2022;12:1012292. Epub 20221025. doi: 10.3389/fonc.2022.1012292. PubMed PMID: 36387197; PubMed Central PMCID: PMC9648663.

41. Ke HL, Li CC, Lee HY, Tu HP, Wei YC, Yeh HC, et al. Prognostic Value of Comorbidity for Patients with Upper Tract Urothelial Carcinoma after Radical Nephroureterectomy. Cancers (Basel). 2022;14(6). Epub 20220312. doi: 10.3390/cancers14061466. PubMed PMID: 35326617; PubMed Central PMCID: PMC8946018.

42. Koterazawa S, Kanno T, Kobori G, Ito K, Nakagawa H, Takahashi T, et al. Clinical outcomes following laparoscopic radical nephroureterectomy in octogenarians. Int J Clin Oncol. 2023;28(1):155-62. Epub 20221121. doi: 10.1007/s10147-022-02269-8. PubMed PMID: 36414826.
